# Metagenomics reveals the abundance and accumulation trend of antibiotic resistance gene profile under long-term no tillage in a rainfed agroecosystem

**DOI:** 10.3389/fmicb.2023.1238708

**Published:** 2023-07-20

**Authors:** Weiyan Wang, Pengfei Shen, Zhiqiang Lu, Fei Mo, Yuncheng Liao, Xiaoxia Wen

**Affiliations:** ^1^College of Agronomy, Northwest A&F University, Yangling, Shaanxi, China; ^2^Key Laboratory of Low-carbon Green Agriculture in Northwestern China, Ministry of Agriculture and Rural Affairs, Yangling, Shaanxi, China; ^3^Key Laboratory of Crop Physi-ecology and Tillage Science in Northwestern Loess Plateau, Ministry of Agriculture, Northwest A&F University, Yangling, Shaanxi, China

**Keywords:** conservation tillage, antibiotic resistance genes, soil drug resistance, microbial metabolic activity, metagenomic sequencing

## Abstract

Widespread soil resistance can seriously endanger sustainable food production and soil health. Conservation tillage is a promising practice for improving soil structure and health. However, the impact of long-term no-tillage on the presence of antibiotic resistance genes in agricultural soils remains unexplored. Based on the long-term (>11 yr) tillage experimental fields that include both conservation tillage practices [no tillage (ZT)] and conventional tillage practices [plough tillage (PT)], we investigated the accumulation trend of antibiotic resistance genes (ARGs) in farmland soils under long-term no-tillage conditions. We aimed to provide a scientific basis for formulating agricultural production strategies to promote ecological environment safety and human health. In comparison to PT, ZT led to a considerable reduction in the relative abundance of both antibiotic resistance genes and antibiotic target gene families in the soil. Furthermore, the abundance of all ARGs were considerably lower in the ZT soil. The classification of drug resistance showed that ZT substantially decreased the relative abundance of Ethambutol (59.97%), β-lactams (44.87%), Fosfomycin (35.82%), Sulfonamides (34.64%), Polymyxins (33.67%), MLSB (32.78%), Chloramphenicol (28.57%), Multi-drug resistance (26.22%), Efflux pump (23.46%), Aminoglycosides (16.79%), Trimethoprim (13.21%), Isoniazid (11.34%), Fluoroquinolone (6.21%) resistance genes, compared to PT soil. In addition, the abundance of the bacterial phyla Proteobacteria, Actinobacteria, Acidobacteria, and Gemmatimonadetes decreased considerably. The Mantel test indicated that long-term ZT practices substantially increased the abundance of beneficial microbial flora and inhibited the enrichment of ARGs in soil by improving soil microbial diversity, metabolic activity, increasing SOC, TN, and available Zn, and decreasing pH. Overall, long-term no-tillage practices inhibit the accumulation of antibiotic resistance genes in farmland soil, which is a promising agricultural management measure to reduce the accumulation risk of soil ARGs.

## 1. Introduction

Antibiotic resistance genes (ARGs), a new type of environmental pollutant, are widely enriched in farmland soils, promoting the spread of drug resistance and seriously affecting soil safety (Huijbers et al., 2015; Rosenkrantz et al., 2019). The soil–plant system is a significant source of exposure to environmental resistance genes, which can spread from the environment to humans (Wang et al., 2022a,b). The food chain is the most direct and main pathway for resistant bacteria and genes to enter the human body in soil–plant systems (Blau et al., 2018). With the rapid development of the global economy, resistance genes in agricultural products can spread rapidly worldwide through food processing, preservation, and global transportation, triggering public safe risks and affecting global health (Nowakowska et al., 2019; Zhu et al., 2019). Therefore, to remove and control ARGs in farmland soils, it is particularly important to clarify the influence of soil management practices on ARGs and their relationship with microbial communities.

Cropping system soil, one of the habitats with the highest microbial diversity on Earth, is a huge reservoir of ARGs (Forsberg et al., 2012; Sanderson et al., 2016; Van Goethem et al., 2018). Internal resistance and exogenous input are the main sources of ARGs in agricultural soil (Riber et al., 2014; Nõlvak et al., 2016). Internal resistance refers to the inherent genome of microorganisms, that can be inherited by subsequent generations. Exogenous input involves the horizontal transfer and enrichment of ARGs directly into the soil through the addition of external sources, such as manure, sludge, and reclaimed water. The composition and abundance of ARGs are closely linked to the microbial community and can be affected by changes in microbial community diversity and composition (Forsberg et al., 2014; Liu et al., 2019; Zhang et al., 2021). Studies have shown that *Actinobacteria* produce the most natural antibiotics, and *Firmicutes* and *Actinobacteria* have been identified as the main groups carrying ARGs (Zhou et al., 2015; Xun et al., 2016). Chen et al. (2016) found that the abundance of tetracyclines ARGs increased as the abundance of *Actinomycete* increased and the abundance of *Chloroflexi* decreased. A study have also shown that the increased abundance of Proteobacteria was accompanied by increasing β-lactam-resistance genes (Wang et al., 2016). In addition, Xiang et al. (2018) and Urra et al. (2019) showed that the decrease of soil microbial α-diversity led to an increase in MLSB, aminoglycosides, β-lactams, amide alcohols, sulfonamides ARGs and transposase gene *tnpA*. In summary, previous studies mainly focuses on manure application, sewage-irrigated, and typically contaminated soils (Achiba et al., 2010; Xie et al., 2018), however, there are no reports on the influence mechanism of tillage management measures on agricultural soil ARGs.

The introduction and enrichment of ARGs in farmland soils are commonly attributed to agricultural practices (Wu et al., 2022). For example, manure application can considerably enrich resistant bacteria and ARGs in agricultural soils (Munk et al., 2018; Xie et al., 2018). Fertilizer application can significantly increase the abundance of important resistance genes, including *intI1*, *tetW*, *tetO*, *sul1*, and *sul2* (Xie et al., 2018). Zhao et al. (2019) found that the abundance of tetracycline- and ampicillin-resistant bacteria in greenhouse soils was substantially higher than that in field soils. Besides the change of soil physical and chemical properties (i.e., pH, soil organic carbon (SOC), total nitrogen (TN), NH_4_^+^, NO_3_^−^, soil texture, etc) may create different niches for bacterial populations and alter the microbial communities, thereby affecting the composition and abundance of ARGs (Dumbrell et al., 2010). In addition, Zhao et al. (2017) found that the concentration of some heavy metals (such as Cu, Zn, Mn, Fe) in soil may also drive the distribution and spread of ARGs in the environment. Conservation tillage, that is, reduced or no tillage with straw return, can decrease soil disturbance and improve its physical and chemical properties, thereby leading to changes in microbial habitats and communities, consequently affecting likely the abundance of ARGs in agricultural soil (Wang et al., 2017, 2021). This practice has been widely used in dry farmlands in northern China (Wang et al., 2020). However, the impact of long-term no-tillage on ARGs in agricultural soil remains unknown, hampering a complete understanding of ARGs in this context.

Therefore, based on a long-term (> 11 yr) conservation tillage experimental field, we investigated the effect on the abundance of ARGs and microbial community composition using shotgun metagenomic sequencing under two different tillage systems, no tillage and plough tillage, in a winter wheat–summer maize rotation system. We hypothesized that long-term no-till application could reduce the abundance and accumulation of ARGs by improving soil properties and microbial community structure. Therefore, three main objectives were established: (1) to analyze the effects of different tillage treatments on the relative abundance of ARGs; (2) to study the contribution of the microbial community to ARGs under long-term no tillage vs. plough tillage; and (3) to determine the correlations between soil variables and changes in ARGs under different tillage treatments.

## 2. Materials and methods

### 2.1. Experimental site and design

This study was conducted at the conservation tillage long-term experimental field (34°16′48″ N and 108°4′12″ E) in the north campus of Northwest A&F University, Yangling, Shaanxi, China. The site is located at the southern part of the Loess Plateau and has a temperate monsoon climate, with high summer temperatures and cold winters, annual average temperature of 12.9°C, and the frost-free period of 221 d. The annual rainfall is approximately 620 mm, and 60–70% of the rainfall occurs between July and October. The soil texture was classified as silty clay (Soil Survey Staff, 2010). The initial soil properties at a depth of 0–20 cm was reported in a previous study (Wang et al., 2021). This is the main winter wheat-summer maize double-cropping area in China.

The long-term tillage experiment in this study began in 2009, with a total of two conservation tillage treatments [i.e., no tillage with straw return (ZT) and conventional tillage plough with straw return (PT)]. In this study, the ZT treatment, the most representative conservation tillage practice, was selected to explore the effect of long-term conservation tillage on ARGs and the driving mechanism that ensures summer maize growth. A randomized block design was adopted with three replicates for each treatment. The plot area was 125 m^2^ (25 × 5 m). Tillage operations were performed before sowing winter wheat and summer maize. The specific tillage steps were as follows: in ZT, to minimize soil disturbance and create a good seedling bed to ensure seedling emergence, a rotary machine was used for shallow rotation with a depth of 5 cm, and then the maize precision seeder was used for sowing; in PT, the plot was ploughed to a depth of 20–30 cm by a mouldboard plough machine, the seedbed was arranged by a rotary tiller, and the maize precision seeder was used for sowing.

Summer maize (cultivar Shandan 609) was sown on 17 June 2018 and harvested on 5 October 2018. The row spacing and plant spacing of summer maize plant were 70 cm and 23 cm, respectively. Wheat straw was crushed in pieces less than 5 cm in length and evenly distributed on the ground after harvest and then tilled and sown. The fertilizers were applied as basal fertilizers; which involved spreading them on the soil surface by hand before tillage. The amount of urea (N, 46%) and diammonium phosphate (N, 18%; P_2_O_5_, 46%) applied was 375 kg hm^−1^.

### 2.2. Soil sampling and physicochemical properties analysis

During the 2018 summer maize growing season, soil samples were collected on the first day after tillage, seedling stage, and silking stage under two tillage treatments, with three replicates. Using the S-type sampling method, five soil cores (0–20 cm depth) were collected from each plot to form a sample, and large rocks and roots were screened using a 2 mm mesh. Representative samples from each plot (0–20 cm depth) were divided into two parts: one was stored at −80°C for metagenomic sequencing; and the other was stored at −20°C for determining soil properties and available metal ion concentrations. In addition, the soil bulk density of 0–10 cm and 10–20 cm was measured by ring knife method.

The soil particle size distribution was analyzed using a Malvern laser particle size analyzer (Malvern Ms 2000, Malvern Instruments Ltd., United Kingdom). The moisture content of the soil samples was determined using the gravimetric method, and soil temperature was determined using a digital thermometer sensor Prober (EC-TP101, Henglu Biological Technology Co., Ltd., China). Soil pH at a water-soil ratio of 1:5 was measured using an electronic pH detector (CyberScan pH 510, Thermo Fisher Scientific, United States). Soil inorganic nitrogen was extracted by 1 mol L^−1^ KCl solution, and then the concentration of NO_3_^−^ and NH_4_^+^ was determined using an AA3 HR Autoanalyzer (Seal Analytical GmbH, Germany). Soil total nitrogen (TN) content was measured using the Kjeldahl digestion method (Sparks, 1996). Soil organic carbon (SOC) content was determined using the potassium dichromate-concentrated sulfuric acid plus heat capacity method (Wang et al., 2021). Microbial biomass carbon (MBC) was calculated from the difference of SOC between chloroform fumigated samples and control samples (Ren et al., 2021). Microbial respiration (MR: CO_2_ flux) was measured by alkali absorption. The metabolic quotient (qCO_2_) = MR/MBC, and microbial quotient (Cmic:Corg) = MBC/SOC (Ren et al., 2021). The concentrations of available iron (Fe), manganese (Mn), copper (Cu) and zinc (Zn) were measured by DTPA leaching atomic absorption spectrophotometry method (Achiba et al., 2010).

### 2.3. DNA extraction

Genomic DNA of soil microorganisms was extracted the FastDNA^®^ SPIN Kit for Soil (MP Biomedicals Co., Ltd., Santa Ana, CA, United States) as per the manufacturer’s instructions. Each soil sample was extracted four times and mixed. DNA concentration and purification were determined using a TBS-380 microfluorometer. DNA integrity was detected using 1% agarose gel electrophoresis.

### 2.4. Shotgun metagenomic sequencing, assembly, and ARGs annotation

Six soil samples were collected at the seedling stage (two tillage practices × with three replicates) and selected for shotgun metagenomic sequencing. M220 Focused-ultrasonicator (Covaris M220, Gene Company Limited, Hong Kong, China) was used to break the microbial genomic DNA into 400 bp fragments. The PE library was constructed using a NEXTFLEX Rapid DNA-Seq Library Building Kit (BioScientific, United States). After bridge PCR amplification, libraries were sequenced using an Illumina HiSeq 3000/4000 platform (Illumina, United States) at Majorbio Bio-Pharm Technology Co., Ltd. Approximately 120 GB original sequences were obtained using shotgun metagenome sequencing with 292,349,780 paired reads. Raw reads were uploaded to the NCBI for Biotechnology Information Sequence Reading Archive database (accession no. PRJNA641749).

The adaptors were stripped using SeqPrep.^1^ Raw sequencing reads were subjected to a series of quality controls using fastp^2^, in which reads with a length < 50 bp, quality value < 20, or having N bases were filtered and removed. IDBA-UD/Megahit^3^ and Newbler^4^ were used to maximize the use of high-quality clean reads for multiple hybrid splicing and assembly (Peng et al., 2012; Li D. H. et al., 2015), contigs > 500 bp in length were retained for gene prediction. MetaGene^5^ was used to predict the ORF of the retained contigs. Genes with nucleic acid lengths >100 bp were selected and translated into amino acid sequences to obtain gene prediction tables for each sample. Subsequently, using SOAPaligner software^6^, high-quality reads and non-redundant gene catalog of each sample were compared (95% identity) to obtain non-redundant gene catalog for subsequent analysis (Fu et al., 2012).

The comprehensive antibiotic resistance database^7^ contains reference genes related to antibiotic resistance in various organisms, genomes, and plasmids that can be used to guide research on drug and antibiotic resistance mechanisms in environmental flora (Martínez et al., 2007; Jia et al., 2016). To obtain antibiotic resistance genes annotation tables, DIAMOND software^8^ was used to compare non-redundant gene catalog with CARD database (*e*-value ≤1e-5) (Buchfink et al., 2021). In this step, we performed functional screening at the Class level of the CARD database, in which we constructed gene subsets of the antibiotic biosynthesis (AB), antibiotic resistance (AR), antibiotic sensitive (AS), and antibiotic target (AT) families, respectively, for subsequent analysis. Then, to assess the abundance of these genes, high-quality sequences from each sample were mapped onto the predicted gene sequences using Salmon (Patro et al., 2017) and the transcripts per kilobase per million mapped reads (TPM) were used to normalize the abundance values in the metagenomes. The data were analyzed on the online tool of Majorbio Cloud Platform^9^ (Ren et al., 2022).

### 2.5. Statistical analysis

Microsoft Office Professional Plus 2021 software (Microsoft Co., Redmond, WA, United States) was used for data collection and calculations. One-way ANOVA was performed using the SPSS software (version 19.0; SPSS Inc., Armonk, NY, United States). The significant differences between the treatments were tested by Tukey’s HSD test (*p* < 0.05). Nonmetric multi-dimensional scaling (NMDS) and redundancy analysis (RDA) were performed using “vegan” packages in the R 3.6.1 for Windows (R Core Team, 2022). Comparative analysis of the two groups were conducted to analyze ARGs with significant difference between ZT and PT using the “stats” package in the R 3.6.1 for Windows. Furthermore, based on Pearson correlation analysis and Mantel test, the relationship between antibiotic resistance genes carrying community and microbial metabolic coefficient and soil properties was analyzed using “cor” function and “vegan” package in the R 3.6.1 for Windows.

## 3. Results

### 3.1. Long-term tillage practice contributes to differences in soil characteristics

Long-term ZT significantly altered soil physical properties, chemical properties, and available soil concentrations of Fe, Mn, Cu, and Zn (*p* < 0.05). Compared with the PT treatment, the ZT treatment greatly increased the fraction of sand and silt, soil moisture, soil bulk in the 0–10 cm layer, SOC, TN, NO_3_^−^, and NH_4_^+^. Conversely, the clay fraction, pH, and soil bulk (10–20 cm layer) were significantly lower in ZT soil than that in PT soil (Table 1). In addition, the available concentrations of Fe, Mn, Cu, and Zn were enriched in the ZT soil for up to a decade and were considerably higher than those in the PT soil (Table 2).

**Table 1:** The physical and chemical properties of topsoil, as affected by the tillage practices.

Tillage	Physical and chemical properties of topsoil										
	Sand (%)	Slit (%)	Clay (%)	pH	TN (g kg^−1^)	SOC (g kg^−1^)	Moisture (%)	NO_3_^−^ (mg kg^−1^)	NH_4_^+^ (mg kg^−1^)	B10 (g cm^−3^)	B20 (g cm^−3^)
The first day after tillage											
Zero-till	0.97 ± 0.02a	64.72 ± 0.10a	34.31 ± 0.12b	7.60 ± 0.04 b	1.35 ± 0.05a	13.41 ± 0.96a	20.76 ± 0.52a	2.93 ± 0.61a	26.19 ± 1.94a	1.30 ± 0.02a	1.30 ± 0.02b
Plow–till	0.88 ± 0.01b	63.45 ± 0.056b	35.68 ± 0.07a	7.89 ± 0.03 a	1.17 ± 0.03b	10.44 ± 0.46b	18.39 ± 1.21b	0.3 ± 0.32b	9.95 ± 2.65b	1.26 ± 0.02b	1.53 ± 0.083a
The seedling stage (Metagenomic sequencing period)											
Zero-till	1.19 ± 0.01a	63.70 ± 0.19b	35.20 ± 0.41b	7.50 ± 0.09 b	1.37 ± 0.04a	11.99 ± 1.89a	17.31 ± 0.64a	8.83 ± 0.82a	4.21 ± 0.56a	1.33 ± 0.03a	1.39 ± 0.02b
Plow–till	0.90 ± 0.04b	62.45 ± 0.24b	36.55 ± 0.24a	7.78 ± 0.07 a	1.18 ± 0.06b	9.63 ± 0.53b	15.37 ± 0.62b	7.33 ± 0.28a	2.03 ± 0.52b	1.29 ± 0.02b	1.54 ± 0.11a
The silking stage											
Zero-till	1.23 ± 0.01a	63.61 ± 0.38a	35.07 ± 0.13b	7.65 ± 0.03 b	1.34 ± 0.12a	13.26 ± 2.50a	14.16 ± 0.68a	0.32 ± 0.04a	2.25 ± 0.30a	1.38 ± 0.01a	1.39 ± 0.05b
Plow–till	0.79 ± 0.03b	62.55 ± 0.22b	36.71 ± 0.23a	7.92 ± 0.05 a	1.15 ± 0.01b	9.66 ± 2.64b	12.03 ± 0.82b	0.18 ± 0.04b	2.03 ± 0.27a	1.31 ± 0.02b	1.55 ± 0.13a
Analysis of variance											
Tillage (T)	**	**	**	**	**	**	**	**	**	*	**
Time (Ti)	ns	ns	ns	**	ns	ns	**	**	**	*	ns
T × Ti	ns	ns	ns	ns	ns	ns	*	**	**	ns	ns

Results are reported as means ± SD (*n* = 3). Different letters within a row indicate significant differences between the treatments by Tukey’s HSD test (*p* < 0.05). * and ** Indicates significant difference at *p* < 0.05, *p* < 0.01, respectively; ns, indicates no significant difference. B10, soil 0 ~ 10 cm bulk density; B10, soil 10 ~ 20 cm bulk density. In addition to soil bulk density, the sampling depth of other soil properties was 0 ~ 20 cm.

**Table 2:** The available concentrations of Fe, Mn, Cu, and Zn, as affected by the tillage practices.

Tillage	Soil microelement concentration (mg kg^−1^)			
	Fe	Mn	Cu	Zn
Zero-till	5.79 ± 0.11 a	20.53 ± 0.98 a	1.03 ± 0.04 a	0.95 ± 0.04 a
Plow–till	5.39 ± 0.15 b	17.95 ± 0.86 b	0.94 ± 0.05 b	0.81 ± 0.1 b
ANOVA results				
Tillage (T)	***	**	**	**

Results are reported as means ± SD (*n* = 3). Different letters within a row indicate significant differences between the treatments by Tukey’s HSD test (*p* < 0.05). ** and *** Indicates significant difference at *p* < 0.01, *p* < 0.001, respectively; ns, indicates no significant difference.

### 3.2. Trends of antibiotic resistance gene families under long-term no tillage vs. plough tillage

**Figure 1:**
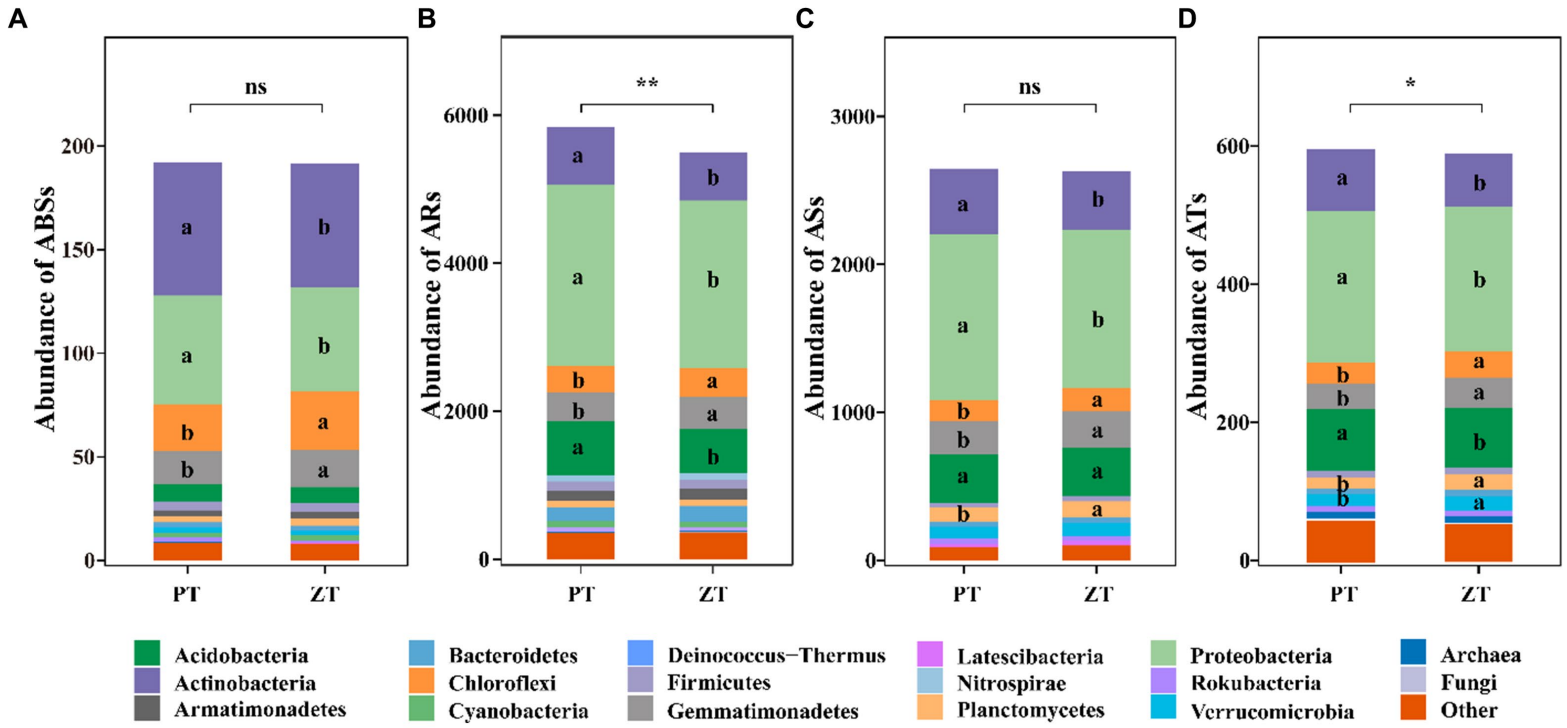
The abundance (TPM: transcripts per kilobase per million mapped reads) of microbial antibiotic biosynthesis (ABSs, **A**), antibiotic resistance (ARs, **B**), antibiotic sensitive (ASs, **C**), and antibiotic target (ATs, **D**) following tillage practices. ZT, no tillage; PT, plough tillage.

**Figure 2:**
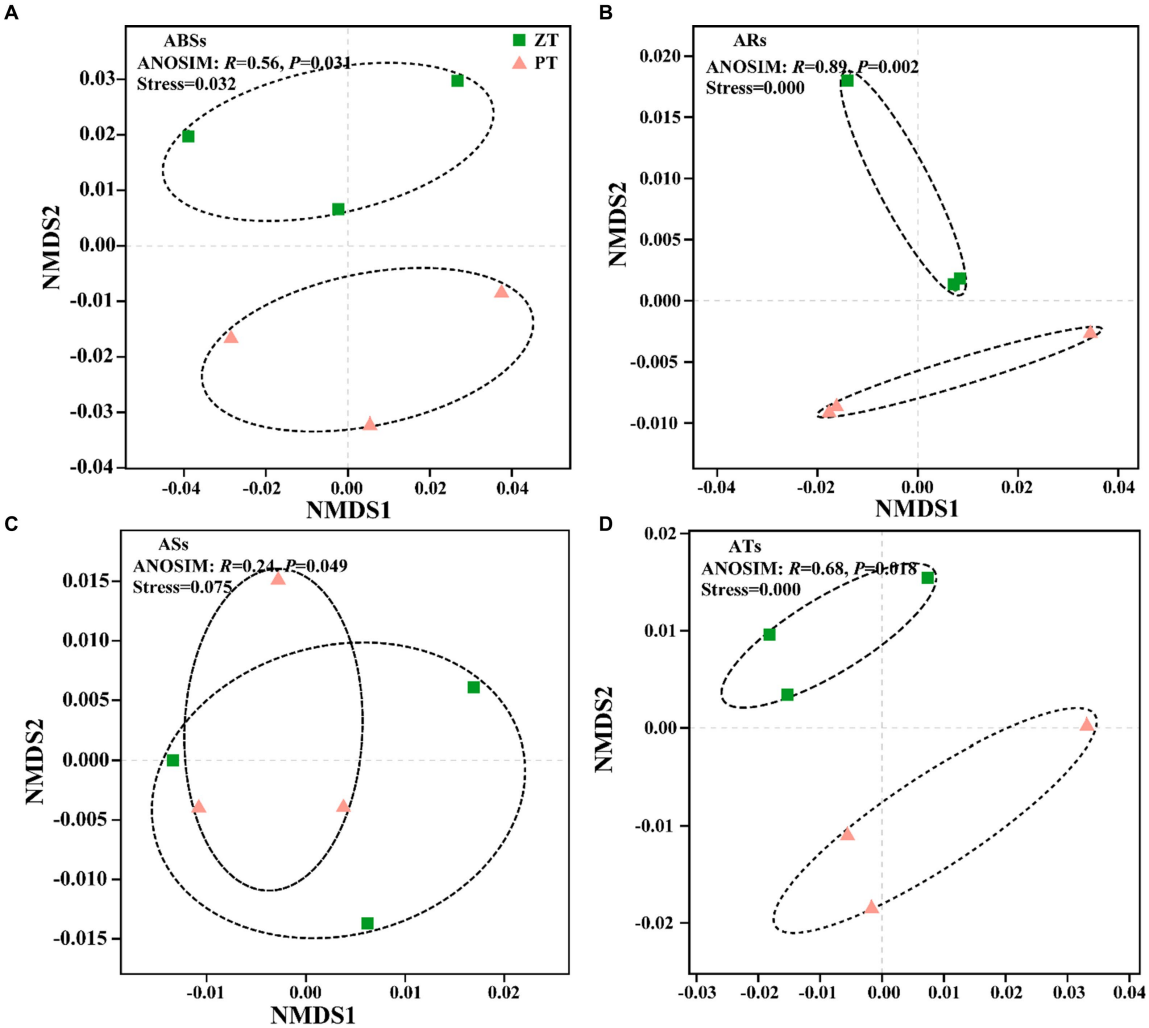
Nonmetric multidimensional scaling of the antibiotic biosynthesis (ABSs, **A**), antibiotic resistance (ARs, **B**), antibiotic sensitive (ASs, **C**), and antibiotic target (ATs, **D**) following tillage practices. ZT, no tillage; PT, plough tillage.

Compared with PT, the application of long-term ZT significantly decreased the abundance of ARs and ATs (Figure 1, *p* < 0.05), along with ABSs and ASs. The NMDS further illustrated significant differences in ABSs (ANOSIM: *r* = 0.56, *p* = 0.031, Figure 2A), ARs (ANOSIM: *r* = 0.89, *p* = 0.002, Figure 2B), ASs (ANOSIM: *r* = 0.24, *p* = 0.049, Figure 2C), and ATs (ANOSIM: *r* = 0.68, *p* = 0.018, Figure 2D) families between the ZT and PT treatments.

A total of 703,162 ARGs were identified from the 6,395,732 non-redundant gene catalogs of the whole metagenome (1.00%, average value), which were matched 13,104 ARO accession and assigned to 434 ARO names in the CARD database. Among these, 93.44, 0.05, and 0.56% were assigned to bacteria, fungi, and archaea, respectively, whereas the remaining 5.95% were unassigned. Moreover, most ABSs, ARs, ASs, and ATs ARGs were largely explained by the bacterial community, which accounted for 95.45% of the ABSs reads (Actinobacteria: 32.25%, Proteobacteria: 26.84%, Chloroflexi: 13.19%, and Gemmatimonadetes: 8.94%, etc), 93.43% of the ARs reads (Proteobacteria: 41.45%, Actinobacteria: 12.81%, and Acidobacteria: 12.34%, etc), 96.24% of the ASs reads (Proteobacteria: 41.49%, Actinobacteria: 15.90%, and Acidobacteria: 12.47%, and Gemmatimonadetes: 8.93%, etc), and 88.65% of the ATs reads (Proteobacteria: 36.16%, Acidobacteria: 14.72%, and Actinobacteria: 14.09%, etc) (Figure 1).

**Figure 3:**
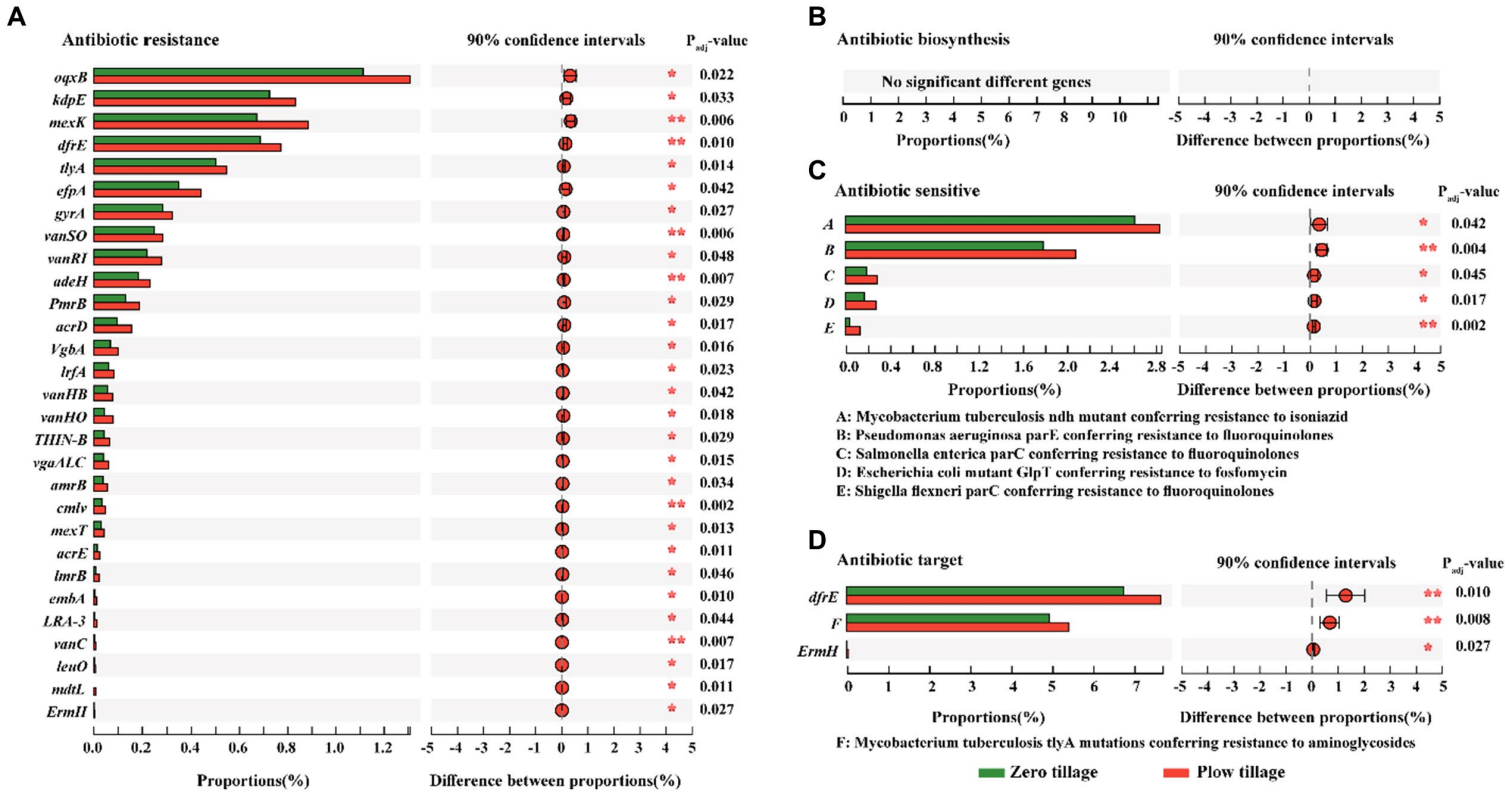
Analysis for significant differences between the groups based on the abundance of antibiotic biosynthesis **(A)**, antibiotic resistance **(B)**, antibiotic sensitive **(C)**, and antibiotic target **(D)**. Gene abundances were based on TPM: transcripts per kilobase per million mapped reads of metagenomic reads. The ordinate represents the names of the genes that encode different enzymes, the abscisic represents the percentage value of a certain gene abundance of the sample, and different colors represent different groups. The right reflected by the abundance response ratios of different gene groups. Error bars represent 90% confidence intervals. *Significant at *p* < 0.05; **significant at *p* < 0.01.

**Figure 4:**
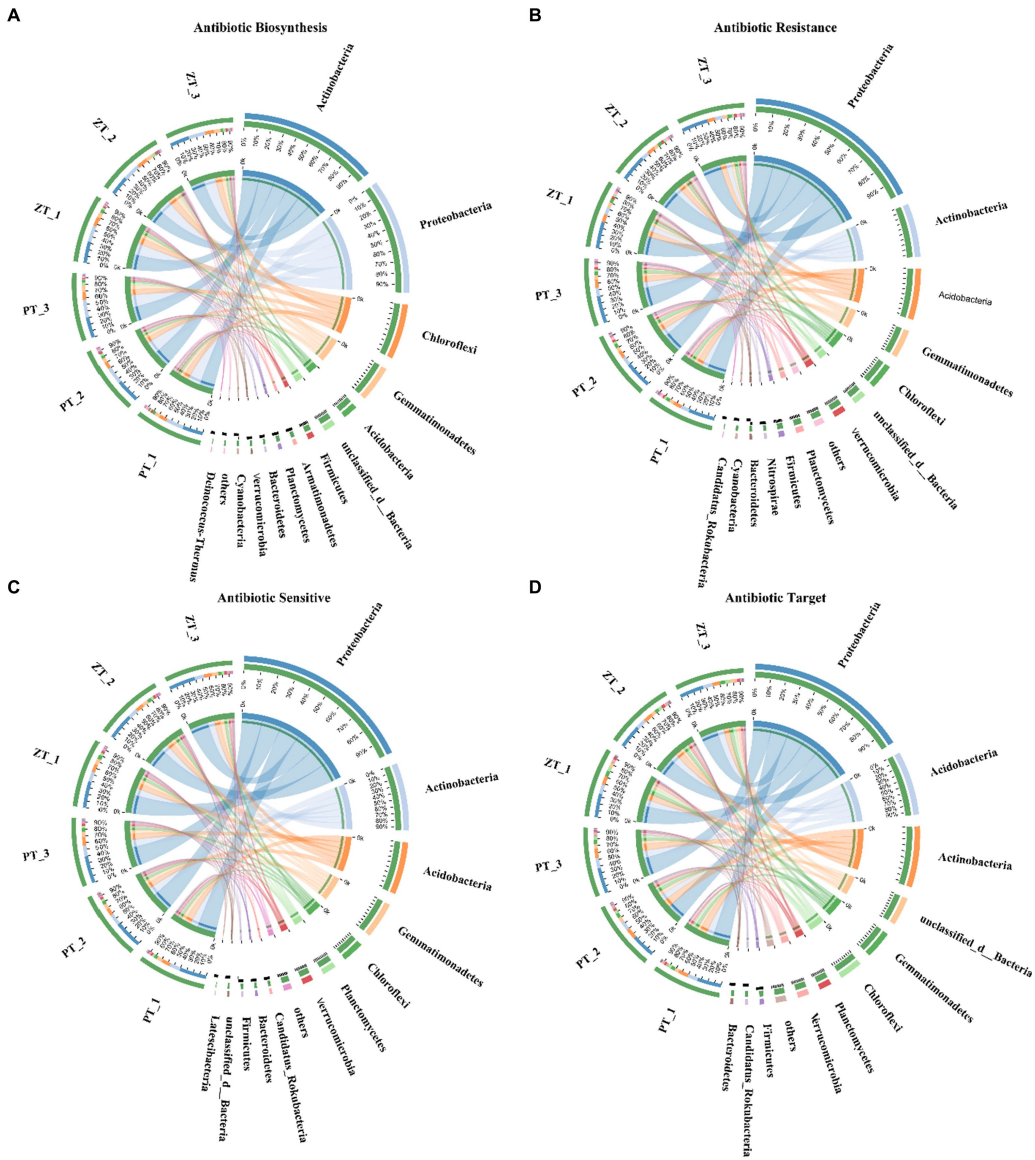
Contribution of microbial (bacterial and fungal) phyla to microbial ARGs genes for the Antibiotic Biosynthesis, Antibiotic Resistance, Antibiotic Sensitive, and Antibiotic Target classes. **(A)** Contribution of microbial (bacterial and fungal) phyla to Antibiotic Biosynthesis. **(B)** Contribution of microbial (bacterial and fungal) phyla to Antibiotic Resistance. **(C)** Contribution of microbial (bacterial and fungal) phyla to Antibiotic Sensitive. **(D)** Contribution of microbial (bacterial and fungal) phyla to Antibiotic Target.

Among the functional groups of ARGs, metagenomic analysis revealed significant differences in the abundance of ARGs between ZT and PT soils (Figure 3). These trends in antibiotic biosynthesis, resistance, sensitivity, and target families were largely assigned to the dominant bacterial phyla, including Proteobacteria, Actinobacteria, Acidobacteria, and Gemmatimonadetes (Figure 4). In particular, Proteobacteria, Actinobacteria, Acdobacteria exhibited a higher number of TMP, which was higher in the PT than in the ZT soil, whereas Gemmatimonadetes and Chloroflexi were significantly lower in the PT than in the ZT soil.

### 3.3. Significant differences of antibiotic resistance gene profile under long-term no tillage vs. plough tillage

For the antibiotic resistance gene family, significantly different gene abundances, such as *oqxB*, *kdpE*, *mexK*, *dfrE*, *tlyA*, etc., were significantly inhibited in ZT soil (Figure 3A). In addition, antibiotic-sensitive and antibiotic target families were enriched in the PT treatment (Figures 3C,D). There were no significant differences in the abundance of antibiotic biosynthesis genes between the two tillage treatments (Figure 3B). These results indicate that long-term ZT can effectively reduce the relative abundance of ARGs.

Furthermore, after analyzing the antibiotics encoded by the significant difference antibiotic resistance genes family, we found that the abundance of *fluoroquinolone*, *efflux pump*, *aminoglycosides*, *isoniazid*, *trimethoprim*, *multi-drug resistance*, *polymyxins*, *fosfomycin*, *MLSB*, *β-lactams*, *chloramphenicol*, *sulfonamides*, *ethambutol* in the two soils was significantly different (Table 3). Compared with PT soil, the abundance of these 13 antibiotic genes was significantly decreased by 6.21, 23.46, 16.79, 11.34, 13.21, 26.22, 33.67, 35.82, 32.78, 44.87, 28.57, 34.64, and 59.97% in ZT soil, respectively, indicating that long-term ZT application has a positive effect on reducing the cumulative risk of antibiotics in agricultural soil.

**Table 3:** The abundance of drug resistance, as affected by the tillage practices.

Drug resistance type	Zero-till	Plow–till	*F*	*P*
Fluoroquinolone	6935.33 ± 168.40 b	7394.67 ± 273.18 a	8.441	0.044
Efflux pump	2259.33 ± 76.38 b	2952.00 ± 203.45 a	30.497	0.005
Aminoglycosides	1189.33 ± 76.27 b	1429.33 ± 51.63 a	20.371	0.011
Isoniazid	1022.00 ± 80.07 b	1152.67 ± 46.88 a	18.363	0.013
Trimethoprim	910.67 ± 15.01 b	1049.33 ± 91.22 a	32.652	0.005
Multi-drug resistance	435.33 ± 22.30 b	590.00 ± 57.58 a	18.820	0.012
Polymyxins	174.67 ± 25.79 b	263.33 ± 19.73 a	22.363	0.009
Fosfomycin	57.33 ± 18.15 b	89.33 ± 9.45 a	31.135	0.005
MLSB	54.67 ± 15.01 b	81.33 ± 9.02 a	20.000	0.011
β-lactams	28.67 ± 2.31 b	52.00 ± 8.00 a	23.558	0.008
Chloramphenicol	30.00 ± 5.29 b	42.00 ± 4.00 a	9.818	0.035
Sulfonamides	2.00 ± 0.00 b	3.06 ± 3.06 a	9.143	0.039
Ethambutol	2.67 ± 1.15 b	6.67 ± 1.15 a	18.000	0.013

Results are reported as means ± SD (*n* = 3). The unit of drug resistance abundance is transcripts per kilobase per million mapped reads (TPM). Different letters within a row indicate significant differences between the treatments by Tukey’s HSD test (*p* < 0.05).

### 3.4. Changes in soil microbial metabolic coefficients following long-term no tillage vs. plough tillage

We found that the long-term application of no-tillage practices can significantly change soil microbial diversity and metabolic coefficients (Table 4). Compared with PT, the soil organic content, microbial diversity, and microbial biomass carbon significantly increased after continuous application (11 yr) of ZT (*p* < 0.05). Moreover, microbial respiration and qCO_2_ decreased significantly with increasing age under no-tillage application (*p* < 0.05). Whereas, the Cmic:Corg ratio increased significantly with ZT application (*p* < 0.05).

**Table 4:** Changes in soil microbial metabolic coefficients following long-term no tillage.

Parameters	Zero-till	Plow–till	*F*	*P*
SOC (g/kg)	13.98 ± 0.56 a	10.83 ± 0.37 b	59.339	<0.001
MBC (mg/kg)	513.18 ± 22.5 a	353.03 ± 8.51 b	177.291	<0.001
Shannon index	6.85 ± 0.03 a	6.74 ± 0.03 b	15.876	0.011
MR (mg/kg)	113.14 ± 1.51 b	149.45 ± 3.37 a	86.450	<0.001
qCO_2_	0.22 ± 0.01 b	0.42 ± 0.02 a	458.68	<0.001
C_mic_: C_org_	36.71 ± 1.28 a	32.61 ± 0.93 b	26.859	0.002

Values are represented as the mean value followed by standard error in parentheses (*n* = 3). Different lowercase letters indicate a significant difference (*p* < 0.05) between different tillage practices, based on a one-way ANOVA followed by Tukey’s HSD test (*p* < 0.05). SOC: soil organic carbon, MBC: microbial biomass carbon; Shannon index: Microbial diversity; MR: microbial respiration; qCO_2_ stands for the metabolic quotient, which was estimated by MR: MBC; C_mic_: C_org_ stands for the microbial quotient, which was estimated by MBC: SOC.

### 3.5. Specific ARGs family responses for microbial metabolic activity and soil properties

**Figure 5:**
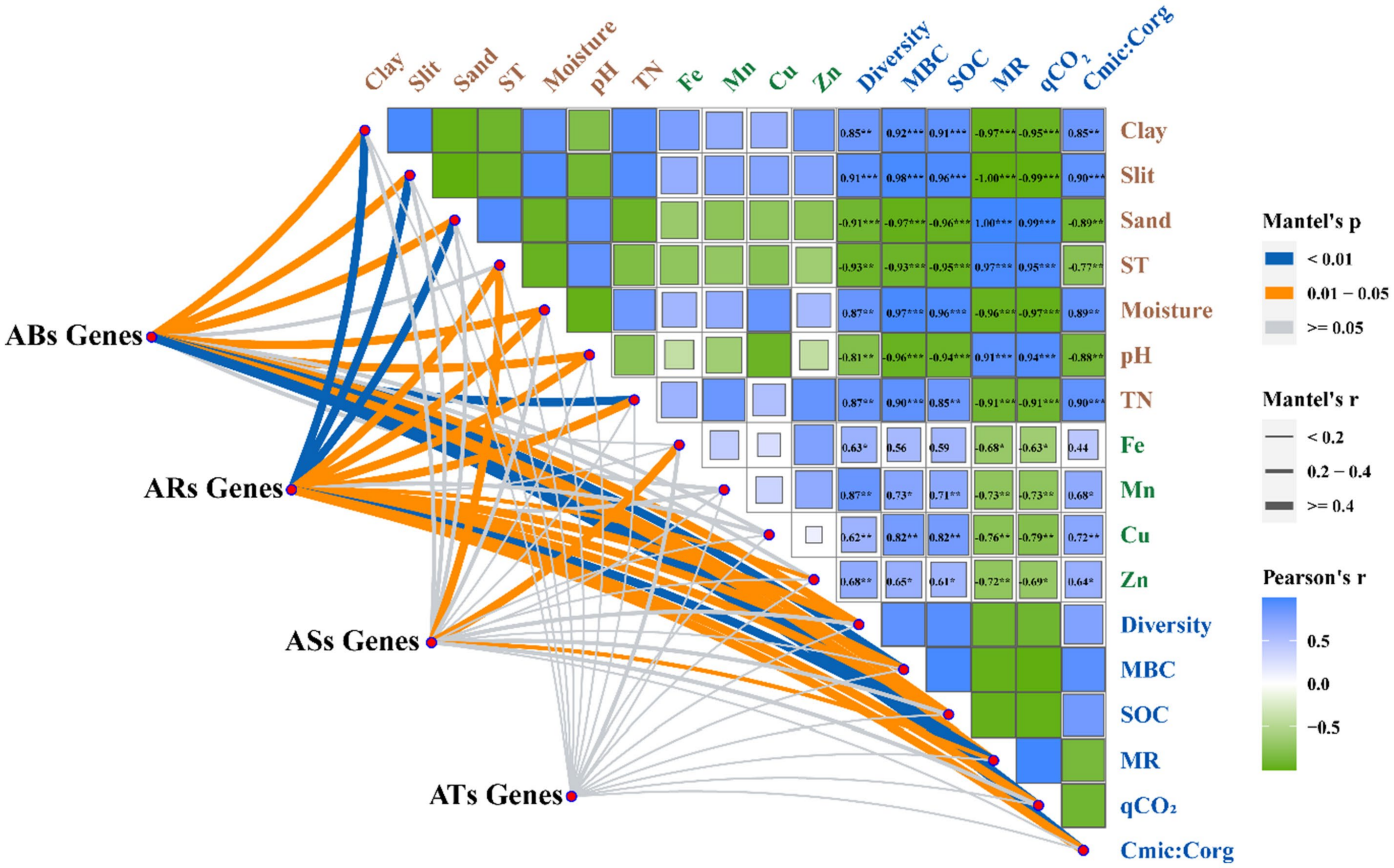
Relationship among the abundance of the ARGs genes involved in antibiotic biosynthesis (ABSs), antibiotic resistance (ARs), antibiotic sensitive (ASs), and antibiotic target (ATs), microbial metabolic activity and soil properties following tillage practices.

The Mantel test indicated that soil texture (clay, silt, and sand), soil moisture, pH, TN, MBC, SOC, and microbial metabolic activity (diversity, MR, qCO_2_, and Cmic:Corg ratio) correlated significantly with various genes of the antibiotic biosynthesis family (Figure 5), in which the effect values of MBC and SOC were the largest. The significantly different genes of the antibiotic resistance family exhibited a strong association with soil texture (i.e., clay, silt, and sand), soil temperature, soil moisture, pH, TN, available Zn, MBC, SOC, and microbial metabolic activity (i.e., diversity, MR, qCO_2_, and Cmic:Corg ratio), in which pH, diversity, MR, and qCO_2_ were the main regulatory factors. Soil temperature, Fe, and MR had a significant impact on the differentially expressed genes of the antibiotic-sensitive family, whereas soil temperature and MR were significantly correlated with differentially expressed genes of the antibiotic target family. However, there was no significant correlation between the antibiotic target gene family and the soil properties.

## 4. Discussion

Agricultural management practices, such as fertilization types, agricultural planting patterns, and planting crops, are assumed to introduce or enrich a large number of ARGs and exert selection pressure on indigenous soil microbial communities (Forsberg et al., 2014; Wang et al., 2017; Li et al., 2022). However, there is a dearth of knowledge regarding how long-term conservation tillage influences the patterns of a wide spectrum of ARGs in dry agricultural soils. In this study, non-metric multidimensional scaling (NMDS) revealed that the soil ARGs clustered into two groups based on the two tillage treatments (Figure 2). These findings indicate that the community structure and abundance of ARGs in the soil were influenced by long-term ZT and PT applications. The total abundance of microbial antibiotic resistance, sensitive, and target genes was observed to be lower after long-term ZT application than after PT (Figure 1). This suggests that the abundance of soil ARGs may decrease with reduced soil disturbance intensity.

Among the general mechanisms of antibiotic resistance, multidrug efflux pumps excluding drugs serve as critical one in multidrug resistance (Hernando-Amado et al., 2016). Our findings indicate that long-term ZT significantly reduced the abundance of soil efflux pump conferring ARGs, with a significant difference of 23.46% compared to PT soil. Simultaneously, a large number and wide distribution of broad-spectrum efflux pumps, such as *oqxB*, *mexK*, *efpA*, *adeH*, *acrD*, *lrfA vgaALC*, *amrB*, *mexT*, *acrE*, *lmrB*, and *mdtL* in no-tillage soils than in plough tillage soils (Supplementary Table S1). These results suggest that long-term ZT can reduce the accumulation of antibiotic-resistant microorganisms by weakening the generalized role of broad-spectrum efflux pumps in protecting cells from multiple antibiotics encountered in the agricultural environment (Zhang et al., 2021). In addition, compared to PT soil, the abundance of *fluoroquinolone, aminoglycosides, isoniazid, trimethoprim, multi-drug resistance, polymyxins, fosfomycin, MLSB, β-lactams, chloramphenicol, sulfonamides, and ethambutol* resistance genes under long ZT soil were also significantly decreased. This may be because long-term ZT creates a soil environment suitable for microbial proliferation, which significantly increases the abundance of beneficial microorganisms, thereby reducing the abundance of antibiotic-resistant microbial hosts (Dunivin and Shade, 2018). These results indicate that long-term ZT application for 11 consecutive years can inhibit the extensive enrichment of ARG abundance in agricultural soil and reduce the spread of resistance (Gullberg et al., 2014; Blanco et al., 2016). This can greatly contribute to the control of soil resistance gene pollution and achieve sustainable agricultural production.

We used mantel test to identify the key factors affecting the accumulation of ARGs, including physical and chemical soil properties, microbial diversity, and microbial metabolic activity (Supplementary Table S2). The conservation tillage has important effects on the accumulation of ARGs in soil by regulating soil microhabitats. Specifically, the abundance of antibiotic biosynthesis, resistance, sensitivity, and target genes was significantly regulated by soil pH (Figure 5). This is consistent with previous studies showing that pH is the most important factor affecting the microbial community and accumulation of antibiotic resistance genes (Rousk et al., 2010; Li et al., 2020). Changes in soil pH led to significant changes in the soil physicochemical properties and microbial communities, resulting in changes in the accumulation of ARGs in the soil (Liu et al., 2017). In addition, mental analysis showed that the SOC content had a significant effect on AGR accumulation (Figure 5). The ARGs in the no-tillage soil were significantly lower than those in the plough tillage soil, mainly because long-term no-tillage formed a carbon-rich microenvironment that was conducive to the balanced proliferation of various microbial populations, thereby inhibiting the niche of the ARGs host population and reducing the accumulation of ARGs in the soil (Forsberg et al., 2014). Besides, our result also found that the concentration of soil available Zn had a significant effect on the Ars family genes, and the concentration of available Fe had a significant effect on the ASs family genes (Figure 5). This was mainly due to long-term no-tillage significantly changed the concentration of available Zn and Fe, which had an effect on soil bacteria and bacteriophages, and thus inhibited the accumulation of ARGs in no-tillage soil (Dickinson et al., 2019).

In addition to the influence of soil properties on ARGs, the diversity and structural composition of the microbial community also significantly affect ARGs (Dunivin and Shade, 2018). Our previous study found that long-term ZT significantly increased soil bacterial and fungal alpha diversity (Wang et al., 2021), which may be another key biological factor in reducing ARGs accumulation in the soil. A higher soil microbial diversity can significantly inhibit the spread of resistance genes (Chen et al., 2019). Previous studies have confirmed that the decrease of α-diversity of soil microbial communities can lead to the increase the abundance of *MLSB*, *aminoglycosides*, *β-lactams*, *amide alcohols*, *sulfonamides* and *tnpA* resistance genes (Han et al., 2018; Urra et al., 2019). These results are consistent with those of this study (Table 3), which may be because long-term ZT increased the content of organic matter and mineral nutrients in soil, thus increasing the niche breadth of microorganisms in the ZT soil (Forsberg et al., 2014), alleviating the selective pressure on microorganisms (Chase and Myers, 2011), and compressing the living space of antibiotic-carrying host microorganisms (Dunivin and Shade, 2018). Furthermore, Forsberg et al. (2014) and Li B. et al., (2015) found robust correlations between bacterial phyla and ARGs, suggesting that the microbial structure composition could be one of the factors affecting the soil resistome. Our previous studies have also shown that long-term ZT application has significant effects on soil bacterial and fungal community structures (Wang et al., 2017, 2021), suggesting that ZT may lead to changes in the abundance and composition of host populations carrying antibiotic resistance genes. For examples, compared with PT, the relative abundance of *Firmicutes* in ZT soils increased significantly, whereas the relative abundance of *Alphaproteobacteria*, *Betaproteobacteria* and *γ-proteobacteria* decreased significantly (Figure 4). The average number of ARGs in the genome of *Proteobacteria* is the largest, and their proliferation can promote the growth and creation of drug-resistant bacteria in the soil (Zhou et al., 2015; Xun et al., 2016). Udikovic-Kolic et al. (2014) found that the alteration of the soil resistome can mainly be attributed to an increase in certain bacterial populations, such as *Pseudomonas* spp., *Janthinobacterium* sp. and *Psychrobacter pulmonis*, which harbor ARGs in farmland soil.

Microbial metabolic activity also had a significant effect on the accumulation of antibiotic resistance genes (Figure 5). The results showed that the long-term ZT soil microbial metabolic activity was significantly higher than that of the PT soil, which may be another key factor in reducing the accumulation of ARGs in long-term ZT soils. This is mainly because the higher the metabolic activity of microorganisms, the higher their versatility and health, thereby occupying a niche and crowding out the living space of antibiotic-carrying host microorganisms (Yang et al., 2022). Su et al. (2015) reported similar findings, demonstrating that soil with stronger microbial metabolic activity and a higher relative balance of community structure composition can inhibit the abundance and activity of pathogenic microorganisms and antibiotic gene hosts more effectively.

## 5. Conclusion

The long-term application of ZT significantly reduced the accumulation of fluoroquinolone, aminoglycosides, isoniazid, trimethoprim, multi-drug resistance, polymyxins, fosfomycin, MLSB, β-lactams, chloramphenicol, sulfonamides, and ethambutol resistance genes under dryland conditions, compared to PT soil. Such antibiotic resistance gene reduction is closely associated with soil properties, microbial community composition, and metabolic activity. Our findings suggest that long-term ZT practices can reduce the accumulation of antibiotic resistance genes through the following mechanisms: decreased pH and increased SOC content are key physicochemical factors that inhibit ARG accumulation in no-tillage soil, while decreased abundance of Proteobacteria, Actinobacteria, and Acidobacteria communities, and increased abundance of Gemmatimonadetes and Chloroflexi communities, as well as microbial metabolic activity, are key biological factors that inhibit the reproduction of antibiotic-resistant host microbes. This implies that long-term no-tillage practices inhibit the accumulation of antibiotic resistance genes in farmland soil, which is a promising agricultural management measure to reduce the accumulation risk of soil ARGs. Therefore, combining conservation tillage with other high-yield and efficient agricultural practices will greatly improve the sustainability of agricultural production and make a significant contribution to human health in future agricultural production. Future research should identify the risks of active antibiotic resistance gene host microbial populations in the agricultural environment at the transcriptome level, based on RNA sequencing, to ensure that ZT is used sustainably.

## Data availability statement

Raw reads were uploaded to the NCBI Sequence Read Archive database (accession no. PRJNA641749).

## Author contributions

WW: conceptualization, investigation, data curation, writing—original draft, review, editing, and funding acquisition. PS and ZL: partial data investigation. FM: conceptualization, writing—reviewing and editing. YL: conceptualization and funding acquisition. XW: conceptualization, funding acquisition, supervision, writing—reviewing and editing. All authors contributed to the article and approved the submitted version.

## Funding

This study was financially supported by the Science and Technology Plan Project of Shaanxi Province (Key Industry Innovation Chain Project) (grant number 2023-ZDLNY-03), Starting Research Fund from the Northwest A&F University (grant number 2452021112), and National Natural Science Foundation of China (grant number 31971860).

## Conflict of interest

The authors declare that the research was conducted in the absence of any commercial or financial relationships that could be construed as a potential conflict of interest.

## Publisher’s note

All claims expressed in this article are solely those of the authors and do not necessarily represent those of their affiliated organizations, or those of the publisher, the editors and the reviewers. Any product that may be evaluated in this article, or claim that may be made by its manufacturer, is not guaranteed or endorsed by the publisher.

## Footnotes

^1^ https://github.com/jstjohn/SeqPrep

^2^ https://github.com/OpenGene/fastp

^3^ https://github.com/voutcn/megahit

^4^ https://ngs.csr.uky.edu/Newbler

^5^ http://metagene.cb.k.u-tokyo.ac.jp

^6^ http://soap.genomics.org.cn

^7^ http://arpcard.Mcmaster.ca

^8^ https://github.com/bbuchfink/diamond

^9^ https://cloud.majorbio.com/page/tools/

## Abbreviations

ZT, No tillage.
PT, Plough tillage
ARG, Antibiotic resistance genes
SOC, Soil organic carbon
TN, Soil total nitrogen
Zn, zinc.
