# Supplementary material for: Metagenomics reveals the abundance and accumulation trend of antibiotic resistance gene profile under long-term no tillage in a rainfed agroecosystem
Source: Front Microbiol. 2023 Jul 20;14:1238708. doi: 10.3389/fmicb.2023.1238708 (PMC10397733; doi:10.3389/fmicb.2023.1238708)
Supplement: Supplementary file 1 [file Table_1.pdf]

Supplementary materials

Metagenomics reveals the abundance and accumulation trend of antibiotic resistance gene profile under long-term no tillage in a rainfed agroecosystem

Weiyan Wang[1,2,3*], Pengfei Shen[1], Zhiqiang Lu[1], Fei Mo[1], Yuncheng Liao[1], Xiaoxia Wen[1,2*]

[1] College of Agronomy, Northwest A&F University, Taicheng Road 3, Yangling, Shaanxi, 712100, PR China

[2] Key Laboratory of Low-carbon Green Agriculture in Northwestern China, Ministry of Agriculture and Rural Affairs, P. R. China

[3] Key Laboratory of Crop Physi-ecology and Tillage Science in Northwestern Loess Plateau, Ministry of Agriculture, Northwest A&F University, Yangling, Shaanxi, 712100, PR China

* **Corresponding author**

Weiyan Wang, wweiyan@nwsuaf.edu.cn; Tel.: +86 29 87082085; Fax.: +86 29 87082085

Xiaoxia Wen, wenxiaoxia6811@163.com and wenxx66@nwsuaf.edu.cn (X.X. Wen); Tel.: +86 29 87082021; Fax.: +86 29 87082021

Table S1. Abundance of microbial phyla encoding the ARGs genes of the Antibiotic Biosynthesis, Antibiotic Resistance, Antibiotic Sensitice, and Antibiotic Target classes after long-term no tillage. Different letters indicate significant differences (ANOVA, P < 0.05,) among different tillage types. *, P<0.05; **, P<0.01.

Group	Taxa	*PT*	*ZT*	*F*	*P*
**Antibiotic Biosynthesis**	**Actinobacteria**	64.14±6.52 a	59.48±3.17 b	9.647	0.036
	**Proteobacteria**	52.39±2.11 a	50.49±2.30 a	1.115	0.351
	**Chloroflexi**	22.6±1.60 b	27.95±2.01 a	13.066	0.022
	**Gemmatimonadetes**	16.17±2.66 b	18.09±2.50 a	7.552	0.049
	**Acidobacteria**	8.13±0.90 a	9.10±0.95 a	1.656	0.268
	**Firmicutes**	4.32±1.33 a	4.29±0.51 a	0.002	0.966
	**Armatimonadetes**	2.89±0.68 a	3.33±1.62 a	0.186	0.689
	**Planctomycetes**	2.87±1.71 b	3.27±0.70 a	7.964	0.048
	**Bacteroidetes**	2.53±1.28 a	2.27±1.45 a	0.056	0.825
	**Verrucomicrobia**	2.55±1.26 a	2.18±0.24 a	5.300	0.083
	**Cyanobacteria**	2.07±0.42 b	2.62±1.10 a	58.989	0.002
	**Deinococcus-Thermus**	2.21±0.57 a	1.30±0.72 a	5.294	0.083
	**Others**	2.40±0.53 a	1.91±0.18 a	2.300	0.240
	**unclassified_d__Bacteria**	6.67±1.85 a	6.48±0.69 a	0.028	0.876
**Antibiotic Resistance**	**Proteobacteria**	2440.77±32.49 a	2363.53±2.74 b	16.831	0.015
	**Actinobacteria**	785.11±8.90 a	700.14±19.77 b	46.094	0.002
	**Acidobacteria**	724.42±58.80 a	705.78±13.52 a	0.286	0.624
	**Gemmatimonadetes**	399.60±6.35 b	431.04±9.51 a	22.679	0.009
	**Chloroflexi**	357.60±10.07 b	388.32±3.71 a	24.569	0.008
	**Verrucomicrobia**	189.45±7.04 b	217.03±5.81 a	27.343	0.006
	**Planctomycetes**	140.07±9.53 a	156.61±17.00 a	2.159	0.216
	**Firmicutes**	123.98±0.67 a	119.51±5.88 a	1.709	0.261
	**Nitrospirae**	84.78±5.49 a	82.59±7.45 a	0.168	0.703
	**Bacteroidetes**	83.37±10.26 a	77.35±2.39 a	0.982	0.378
	**Cyanobacteria**	78.56±3.86 a	71.31±2.28 b	7.865	0.049
	**Candidatus_Rokubacteria**	60.02±3.11 a	50.03±1.78 b	23.277	0.008
	**Others**	168.23±4.24 a	175.03±7.84 a	1.745	0.257
	**unclassified_d__Bacteria**	208.21±5.85 a	209.42±6.46 a	0.058	0.821
**Antibiotic Sensitice**	**Proteobacteria**	1119.77±28.19 a	1070.48±24 a	5.318	0.082
	**Actinobacteria**	444.00±16.09 a	395.02±6.81 b	23.574	0.008
	**Acidobacteria**	329.53±30.91 a	328.47±4.89 a	0.003	0.956
	**Gemmatimonadetes**	225.45±6.35 b	245.68±11.11 a	7.500	0.042
	**Chloroflexi**	140.39±7.28 b	155.63±15.37 a	7.970	0.048
	**Planctomycetes**	92.65±4.25 b	110.71±3.69 a	30.907	0.005
	**Verrucomicrobia**	76.62±8.09 a	88.01±1.15 a	5.820	0.073
	**Candidatus_Rokubacteria**	38.48±3.54 a	33.44±1.86 b	7.389	0.049
	**Bacteroidetes**	36.14±1.78 a	34.25±0.82 b	8.359	0.045
	**Firmicutes**	32.13±2.92 b	36.42±0.74 a	32.781	0.005
	**Latescibacteria**	19.15±2.77 b	27.71±0.13 a	28.508	0.006
	**Other**	65.52±4.77 a	72.45±4.73 a	0.265	0.634
	**unclassified_d__unclassified**	30.61±3.71 a	32.79±6.29 a	6.548	0.063
**Antibiotic Target**	**Proteobacteria**	219.21±8.53 a	209.19±5.33 a	2.974	0.160
	**Acidobacteria**	88.45±6.68 a	85.95±1.45 a	0.404	0.560
	**Actinobacteria**	90.56±5.04 a	76.4±7.82 b	6.948	0.048
	**Gemmatimonadetes**	36.78±2.78 b	43.89±7.43 a	14.461	0.019
	**Chloroflexi**	30.76±2.6 a	38.27±3.55 a	8.747	0.042
	**Planctomycetes**	16.7±5.31 a	23.46±5.65 a	2.284	0.205
	**Verrucomicrobia**	17.11±0.60 b	20.96±1.48 a	17.317	0.014
	**Firmicutes**	9.70±2.01 a	9.68±0.36 a	0.000	0.986
	**Candidatus_Rokubacteria**	9.20±0.59 a	7.36±0.93 b	5.381	0.041
	**Bacteroidetes**	7.66±2.04 a	8.88±1.17 a	0.800	0.422
	**Other**	19.09±0.28 a	17.04±2.44 a	2.085	0.222
	**unclassified_d__Bacteria**	43.07±5.02 a	38.72±1.77 a	2.004	0.230

Table S2. Mantel test results

Factor	ARGs type							
	ABs		ARs		ASs		ATs	
	Statistic	Significance	Statistic	Significance	Statistic	Significance	Statistic	Significance
Clay	0.505	0.021*	0.692	0.003**	0.304	0.075	0.110	0.223
Slit	0.598	0.011*	0.662	0.002**	0.236	0.088	0.004	0.407
Sand	0.597	0.013*	0.662	0.008**	0.243	0.100	0.014	0.373
ST	0.350	0.047*	0.818	0.014*	0.493	0.026*	0.026	0.393
Moisture	0.487	0.023*	0.813	0.023*	0.324	0.061	0.006	0.415
pH	0.479	0.028*	0.948	0.005**	0.128	0.198	0.112	0.688
TN	0.591	0.010**	0.801	0.016*	0.145	0.179	0.006	0.435
Fe	0.135	0.687	0.157	0.075	0.494	0.033*	0.294	0.156
Mn	0.287	0.090	0.357	0.068	0.094	0.293	0.030	0.476
Cu	0.219	0.177	0.379	0.043*	0.194	0.216	0.078	0.608
Zn	0.332	0.056	0.400	0.022*	0.032	0.425	0.176	0.171
Diversity	0.437	0.028*	0.941	0.009**	0.200	0.160	0.148	0.061
MBC	0.631	0.006**	0.896	0.019*	0.159	0.134	0.087	0.629
SOC	0.608	0.002**	0.864	0.026*	0.222	0.102	0.062	0.527
MR	0.571	0.025*	0.952	0.006**	0.331	0.047*	0.038	0.334
qCO2	0.605	0.008**	0.934	0.023*	0.266	0.056	0.019	0.471
Cmin:Corg	0.625	0.004**	0.669	0.021*	0.039	0.368	0.118	0.716
